# Clinical observation of the therapeutic effects of pegylated recombinant human granulocyte colony-stimulating factor in patients with concurrent chemoradiotherapy-induced grade IV neutropenia

**DOI:** 10.3892/etm.2014.2160

**Published:** 2014-12-30

**Authors:** FENG-PENG WU, JUN WANG, HUI WANG, NA LI, YIN GUO, YUN-JIE CHENG, QING LIU, XIANG-RAN YANG

**Affiliations:** Department of Radiotherapy, Fourth Hospital of Hebei Medical University, Shijiazhuang, Hebei 050011, P.R. China

**Keywords:** pegylated recombinant human granulocyte colony-stimulating factor, concurrent chemoradiotherapy, grade IV neutropenia

## Abstract

The aim of the present study was to investigate the efficacy and side-effects of preventive treatment with pegylated recombinant human granulocyte colony-stimulating factor (PEG-rhG-CSF) on concurrent chemoradiotherapy-induced grade IV neutropenia and to provide a rational basis for its clinical application. A total of 114 patients with concurrent chemoradiotherapy-induced grade IV neutropenia were enrolled. A randomized approach was used to divide the patients into an experimental group and a control group. The experimental group included three subgroups, namely a P-50 group, P-100 group and P + R group. The P-50 group had 42 cases, which were given a single 50-μg/kg subcutaneous injection of PEG-rhG-CSF. The P-100 group had 30 cases, which received a single 100-μg/kg subcutaneous injection of PEG-rhG-CSF. The P + R group comprised 22 cases, which were given a single 50-μg/kg subcutaneous injection of PEG-rhG-CSF and rhG-CSF 5 μg/kg/day; when the absolute neutrophil count (ANC) was ≥2.0×10^9^/l, the administration of rhG-CSF was stopped. The control group (RC group) comprised 20 patients, who received rhG-CSF 5 μg/kg/day by subcutaneous injection until the ANC was ≥2.0×10^9^/l. Changes in the neutrophil proliferation rate and ANC values over time, the neutropenic symptom remission time and incidence of adverse drug reactions were analyzed statistically in each group of patients. In the experimental group, the neutrophil proliferation rate and ANC values were significantly higher than those in the control group; the clinical effects began 12–24 h after treatment in the experimental group, and indicated that the treatment improved neutropenia in ~48 h after treatment. There was no significant difference in the neutrophil proliferation rate and ANC values between the P-50 and P+R groups. In the experimental group, the remission time of neutropenia-induced fever and muscle pain after administration was significantly shorter than that in the control group, with a statistically significant difference (P<0.05). The adverse drug reaction rates showed no significant difference between the experimental group and the control group. PEG-rhG-CSF had good efficacy and safety in the treatment of concurrent chemotherapy-induced grade IV neutropenia. For the treatment of concurrent chemotherapy-induced grade IV neutropenia, a single subcutaneous injection of 50 μg/kg PEG-rhG-CSF is the recommended dose. The effects begin at 12–24 h; if the ANC values are not significantly improved during this time, no supplementary administration of rhG-CSF is necessary.

## Introduction

Neutropenia is a common clinical complication of chemotherapy in cancer patients. It is also an important factor that delays the course of standard treatments in patients. Recombinant human granulocyte colony-stimulating factor (rhG-CSF) is an effective drug for the treatment of chemotherapy-induced neutropenia. However, for patients with grade IV neutropenia, multiple rhG-CSF treatments are usually required. This is likely to extend the antitumor treatment period and increase physical and mental stress in patients. Pegylated recombinant human granulocyte colony-stimulating factor (PEG-rhG-CSF) is rhG-CSF chemically modified by a single methoxy polyethylene glycol group; it is able to alleviate neutropenia with a single dose (1,2) However, due to the short time that it has been used in China, oncologists have many questions about the use, dosage and safety of this therapy in the treatment of patients with grade IV neutropenia. The questions concern whether the same single dose of PEG-rhG-CSF should be used in all patients; whether PEG-rhG-CSF should be added if the neutropenia is not improved in the short term; and whether the side-effects of PEG-rhG-CSF are significantly increased compared with those of rhG-CSF due its greater molecular weight. The present study analyzed the efficacy and safety of PEG-rhG-CSF in 114 patients with concurrent chemoradiotherapy-induced grade IV neutropenia. The results may provide a basis for the clinical application of PEG-rhG-CSF.

## Materials and methods

### General information

From April 2012 to July 2013, a total of 114 patients with concurrent chemoradiotherapy-induced grade IV neutropenia were enrolled at the Radiotherapy Department of the Fourth Hospital of Hebei Medical University (Shijiazhuang, China). Of these, 69 cases were male and 45 cases were female. The patients were aged 35 to 70 years with a median age of 52 years. All patients were in general good condition with a Karnofsky Performance Status score ≥60 points. Blood diseases, bone metastasis, previous history of chemotherapy and radiotherapy were not found, and the liver and kidney function was normal. There were 62 cases of esophageal cancer; the treatment program was three-dimensional conformal radiotherapy/intensity-modulated radiation therapy (IMRT), the total dose was PTV60-66Gy/30 fractions (f), 1 f/day for 5 days; 43 cases had the LFP regimen (leucovorin, fluorouracil and cisplatin) and 19 cases had the DC regimen (docetaxel and cisplatin) for chemotherapy. In addition, there were 52 cases of lung cancer; the treatment program was three-dimensional conformal radiotherapy/IMRT at the same dosage used for esophageal cancer; 28 cases had the EP regimen (etoposide and cisplatin) and 23 cases had the DC regimen for chemotherapy. Single or combined symptoms of fever, muscle pain, fatigue and digestive disorders were present in 83 patients. The study was approved by the Medical Ethics committee of the fourth Medical College of Hebei University (Hebei, China).

### Experimental and treatment groups

According to the World Health Organization grading standards for common adverse reactions of anticancer drugs (3), patients were diagnosed with grade IV neutropenia when the absolute neutrophil count (ANC) was <0.5×10^9^/l. A randomized approach was used to divide the patients into an experimental group and a control group. The experimental group included three subgroups, namely the P-50, P-100 and P + R groups. The P-50 group contained 42 cases, which were given a single 50 μg/kg subcutaneous injection of PEG-rhG-CSF (Jinyouli™; Shijiazhuang Pharmaceutical Group Co., Ltd), Shijiazhuang, China). The P-100 group contained 30 cases, which received a single 100-μg/kg subcutaneous injection of PEG-rhG-CSF. The P + R group contained 22 cases, which were given a single 50-μg/kg subcutaneous injection of PEG-rhG-CSF and 5 μg/kg/day rhG-CSF (Lishengsu™; Beijing SL Pharmaceutical Co., Ltd. Beijing, China); when the ANC was ≥2.0×10^9^/l, the administration of rhG-CSF was stopped. The control group (RC group) comprised 20 patients who received rhG-CSF 5 μg/kg/day by subcutaneous injection until the ANC was ≥2.0×10^9^/l. All patients were given prophylactic anti-inflammatory treatment, based on clinical symptoms, and also received symptomatic and supportive treatment. All patients enrolled voluntarily and provided signed informed consent.

### Detection indices

All patients received a blood routine test to detect the blood neutrophil count (ANC values) at 12, 24, 36, 48, 72, 96, 120 and 144 h after the first application of PEG-rhG-CSF or rhG-CSF. The changes in the neutrophil proliferation rate and ANC values occurring over time after treatment initiation were documented for statistical analysis. The neutrophil proliferation rate was calculated as follows: Neutrophil proliferation rate (%) = (current ANC value/previous ANC value) × 100. For patients with neutropenia-induced adverse symptoms in each group, the symptom remission time after treatment was recorded for statistical analysis. For patients without neutropenia-induced adverse symptoms prior to treatment, the incidence of drug reactions after treatment was recorded for statistical analysis.

### Statistical methods

SPSS statistical software, version 17.0 (SPSS, Inc., Chicago, IL, USA), was used for statistical analysis of experimental data. The neutrophil proliferation rate and ANC values at each time point were compared using analysis of variance of repeated measured data. For patients with neutropenia-induced adverse symptoms, the symptom remission times after treatment were compared with single factor analysis of variance. For patients without neutropenia-induced adverse symptoms prior to treatment, the incidence of drug reactions after treatment were compared by χ^2^ test.

## Results

### Neutrophil proliferation rate changes with time after treatment

**Figure 1:**
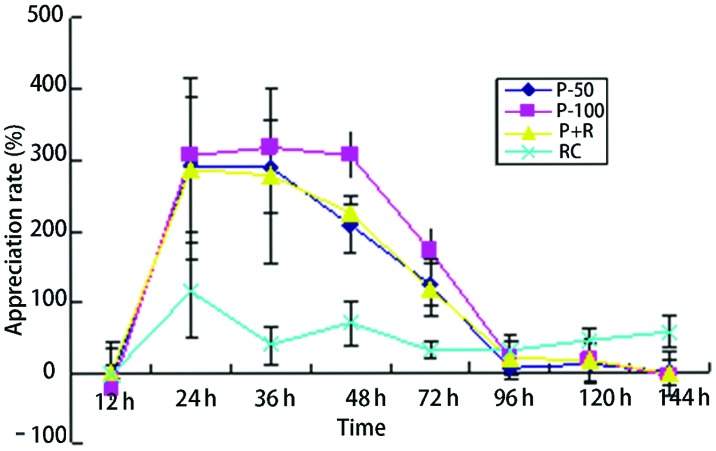
Changes in the proliferation rate of neutrophils over time in patients with grade IV neutropenia following treatment (%). P-50, treated with a single 50-μg/kg subcutaneous injection of PEG-rhG-CSF; P-100, treated with a single 100-μg/kg subcutaneous injection of PEG-rhG-CSF; P + R, treated with a single 50-μg/kg subcutaneous injection of PEG-rhG-CSF and 5 μg/kg/day rhG-CSF; RC, treated with 5 μg/kg/day rhG-CSF. PEG, pegylated; rhG-CSF, recombinant human granulocyte colony-stimulating factor.

In the experimental group, the neutrophil proliferation rate was significantly higher than that in the control group, and the difference was statistically significant (P<0.05). In each experimental subgroup, the neutrophil proliferation rate showed no significant difference between each pair of subgroups (P>0.05). Between the experimental subgroups and the control group, the pairwise comparisons showed statistically significant differences (P<0.05). At 12 h after administration in each group, the mean neutrophil proliferation rates were negative values; in the experimental group, the proliferation rate peaks in the P-50 and P + R groups occurred 24–36 h after treatment initiation. The P-100 peak proliferative rate occurred 24–48 h after treatment. At 144 h after treatment initiation, the mean neutrophil proliferation rates were negative values in each experimental group (Table I, Fig. 1).

### ANC values changes over time after treatment

**Figure 2:**
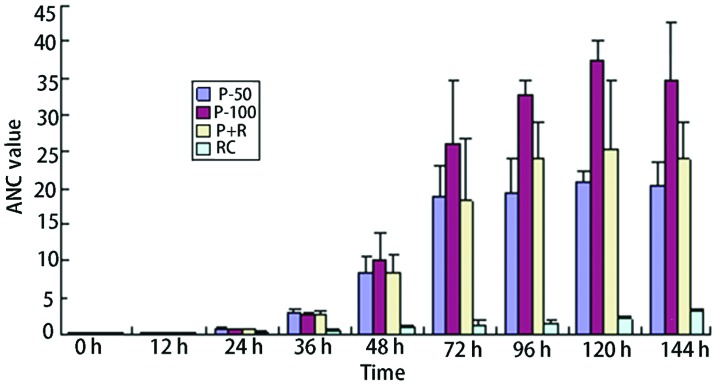
Changes in ANC values over time after treatment in patients with grade IV neutropenia following treatment (x10^9^). P-50, treated with a single 50-μg/kg subcutaneous injection of PEG-rhG-CSF; P-100, treated with a single 100-μg/kg subcutaneous injection of PEG-rhG-CSF; P + R, treated with a single 50-μg/kg subcutaneous injection of PEG-rhG-CSF and 5 μg/kg/day rhG-CSF; RC, treated with 5 μg/kg/day rhG-CSF. ANC, absolute neutrophil count. PEG, pegylated; rhG-CSF, recombinant human granulocyte colony-stimulating factor.

In the experimental group, the ANC values were significantly higher than those in the control group; the differences were statistically significant (P<0.05). At 12 h after the initial drug administration, the blood routine test showed that the mean ANC values had decreased in the patients in each group; the proliferative effect began 12–24 h after the administration of medication. The ANC values in the P-50 and P + R groups showed no significant differences, whereas the P-100 group exhibited statistically significant differences from the P-50 and P + R groups. At 36 h after the initiation of treatment, all three subgroups of the experimental group basically achieved the clinical purpose of completely improving the neutropenia. However, 120 h after the initial administration of medication, ANC values reached 2.1×10^9^/l in the control group; the average amount of rhG-CSF required was 1,650 μg/kg. (Table II, Fig. 2).

### Comparison of the remission time of neutropenia-induced symptoms in the experimental and control groups

In the experimental group, the remission time of neutropenia-induced fever and muscle pain after administration was significantly shorter than the corresponding time in the control group; there was a statistically significant difference between these groups (P<0.05). However, no significant difference was observed in the remission time of neutropenia-induced fatigue and gastrointestinal symptoms between these two groups (Table III).

### Incidence of adverse drug reactions in patients with no neutropenia-induced adverse clinical symptoms pretherapy

The simultaneous or separate incidence of fever, muscle pain, skin rashes, gastrointestinal reaction and other symptoms was 25.3% in the experimental group and 24% in the control group following treatment; there was no statistically significant difference between the two groups (P>0.05).

## Discussion

Numerous studies have confirmed that the modification of protein drugs with polyethylene glycol can create a modified drug with better biological activity and a longer half-life. When compared with rhG-CSF, the polyethylene glycol-modified derivative PEG-rhG-CSF has similar efficacy and safety. It also has a long half-life and a self-regulation effect on blood concentration (4–7). In developed countries such as those in Europe and the USA, PEG-rhG-CSF is mainly used in the preventive treatment of chemotherapy-induced non-marrow-derived neutropenia. In China, the Cancer Hospital of Chinese Academy of Medical Sciences led the research program for domestic PEG-rhG-CSF (Jinyouli), and completed a phase III clinical trial in the prophylactic treatment of chemotherapy-induced neutropenia. The results demonstrated that the efficacy and adverse reactions of single PEG-rhG-CSF administration were similar to those of repeated administration of rhG-CSF (8–9). Given the social and economic factors in China, certain difficulties remain in PEG-rhG-CSF application for preventive treatment in relatively underdeveloped areas. As the drug has been used in China only for a short time, oncologists have questions concerning its use in the salvage treatment of chemotherapy-induced non-marrow-derived neutropenia.

The present study included 114 patients with grade IV chemotherapy-induced neutropenia. The results showed that PEG-rhG-CSF and rhG-CSF both had a beneficial effect on neutrophils. These and other study results are consistent in indicating that PEG-rhG-CSF can be applied as a preventive therapy for chemotherapy-induced neutropenia (9–11). The positive results may also be associated with the fact that the patients in the present study were all chest cancer patients, and the radiation treatment used three-dimensional conformal intensity-modulated technology to better reduce the radiation dose of neighboring flat bones and irregular bones, and therefore, the effects of radiation on the proliferation of bone marrow. The 114 patients received different radiotherapy doses and chemotherapy plans. However, all patients enrolled in the pre-experiment had been confirmed to have no signs of bone metastases. Therefore, the grade IV neutropenia was considered as concurrent chemoradiotherapy-induced non-myeloid-derived neutropenia. In the three subgroups of the experimental group, the neutrophil proliferation rate and ANC values were significantly higher than those in the control group. ANC values in the experimental group increased to the normal range 48 h after treatment, whereas those in the control group only reached 2.0×10^9^/l 120 h after treatment initiation, indicating that a single dose of PEG-rhG-CSF had greater effects on the proliferation of neutrophils than multiple doses of rhG-CSF. PEG-rhG-CSF also significantly shortened the time taken for chemoradiotherapy-induced myeloid-derived non-neutropenia to be improved, and so can reduce the clinical risk of neutropenia.

In the three subgroups of the experimental group, the neutrophil proliferation rates did not show significantly statistical difference. In terms of ANC values, the P-50 group showed no significant difference from the P + R group, and these two groups were statistically different from the P-100 group. In the P-100, P-50 and P + R groups, symptoms of neutropenia were improved 48 h after treatment, so the purpose of clinical treatment was achieved. The difference between the P-100 group and the latter two was that the ANC values of the P-100 group increased more significantly 72 h after treatment. Accordingly, it is proposed that oncologists should use a single subcutaneous dose of 50 μg/kg PEG-rhG-CSF in the treatment of grade IV non-marrow-derived neutropenia to achieve the therapeutic purpose. It is noteworthy that in the experimental and control groups, 12 h after dosing, the neutrophil proliferation rates were negative and ANC mean values showed a downward trend. The reason may be as follows: when grade IV neutropenia was found in certain patients, the ANC values had not dropped to the lowest point induced by the toxicity of chemotherapy; it took some time for rhG-CSF to stimulate the differentiation and maturation of granulocytes. Therefore, the blood ANC values dropped shortly following the injection of PEG-rhG-CSF or rhG-CSF instead of rising. At 24 h after medication, the three experimental subgroups exhibited peak neutrophil proliferation rates, and at 36 h, the ANC mean values all exceeded 2.0×10^9^/l. It is proposed that the blood routine test should be performed 12 h after the application of PEG-rhG-CSF; even if the ANC shows no significant increase, no supplementary administration of rhG-CSF is necessary and the focus should be on changes in ANC values 24–48 h after dosing.

The mean time required to alleviate the neutropenic fever and muscle pain in experimental group was 30 h, compared with 72 and 59 h, respectively, in the control group. This is consistent with the time at which ANC mean values reached 2.0×10^9^/l in the two groups of patients. The times in the two groups were significantly different, indicating that PEG-rhG-CSF had advantages over rhG-CSF in relieving the symptoms of fever and muscle pain caused by neutropenia. In alleviating fatigue and digestive symptoms, PEG-rhG-CSF showed significant advantages over rhG-CSF. This may be due to physical weakness and intestinal dysfunction in certain patients following concurrent chemoradiotherapy.

In terms of safety, the incidence of fever, skeletal muscle pain, skin rashes, gastrointestinal reactions and other symptoms was 25.3% in the experimental group and 24% in the control group; the difference was not statistically significant. The results were comparable with those in the study by Lan *et al* (12). Accordingly the authors of the present study consider that PEG-rhG-CSF is clinically a safe and reliable drug used in patients with non-myeloid-derived neutropenia.

In conclusion, this study showed that PEG-rhG-CSF can be used in concurrent radiotherapy-induced grade IV neutropenia preventive therapy. It demonstrated similar clinical safety to rhG-CSF and significant advantageous effects. A single dose of PEG-rhG-CSF can improve neutropenia and some secondary symptoms, ensure the course of antitumor therapy in these patients, and reduce the pain of repeated rhG-CSF injections, indicating that is has good prospects for the future.

## Figures and Tables

**Table I:** Changes in the neutrophil proliferation rate in patients with grade IV neutropenia over time after treatment (%).

		Time after treatment									
				
Group	n	12 h	24 h	36 h	48 h	72 h	96 h	120 h	144 h	F-statistic	P-value
P-50	42	−9.82±38.37	291.00±42.47	289.54±43.6	207.64±34.47	122.43±30.18	2.97±25.16	9.06±23.92	−2.30±8.41		
P-100	30	−22.83±43.49	304.85±96.34	317.54±62.33	305.99±39.03	171.60±30.90	18.40±8.32	38.23±28.41	−7.67±17.64		
P + R	22	−1.04±10.89	287.12±127.19	277.60±123.78	223.66±10.69	117.32±40.62	19.36±33.39	16.34±31.15	−4.90±31.19		
RC	20	−4.26±47.43	114.84±66.48	37.52±26.40	67.98±32.40	30.14±13.17	30.08±13.81	43.92±16.63	54.92±22.53	12.94	0.002

P-50, treated with a single 50-μg/kg subcutaneous injection of PEG-rhG-CSF; P-100, treated with a single 100-μg/kg subcutaneous injection of PEG-rhG-CSF; P + R, treated with a single 50-μg/kg subcutaneous injection of PEG-rhG-CSF and 5 μg/kg/day rhG-CSF; RC, treated with 5 μg/kg/day rhG-CSF. PEG, pegylated; rhG-CSF, recombinant human granulocyte colony-stimulating factor. There was no pairwise significant difference among the three groups of patients treated with PEG-rhG-CSF (P>0.05); significant differences were observed after 24 h when these three groups were compared with the RC group (P<0.05). The values show the mean ± standard deviation.

**Table II:** Changes in ANC values in patients with grade IV neutropenia over time after (×10^9^).

		Time after treatment initiation										
				
Group	n	0 h	12 h	24 h	36 h	48 h	72 h	96 h	120 h	144 h	F-statistic	P-value
P-50	42	0.22±0.11	0.17±0.05	0.73±0.18	2.73±0.83	8.41±2.02	18.67±4.32	19.32±4.49	20.96±1.20	20.47±3.03		
P-100	30	0.26±0.08	0.19±0.04	0.60±0.17	2.51±0.64	10.19±3.64	26.05±8.54	32.71±2.04	37.61±2.44	34.72±8.04		
P+R	22	0.21±0.14	0.19±0.10	0.69±0.13	2.60±0.65	8.42±2.46	18.27±8.51	24.07±5.10	25.22±9.48	23.98±5.01		
RC	20	0.19±0.12	0.18±0.09	0.34±0.08	0.51±0.16	0.86±0.41	1.12±0.87	1.46±0.52	2.10±0.13	3.06±0.28	65.23	<0.01

P-50, treated with a single 50 μg/kg subcutaneous injection of PEG-rhG-CSF; P-100, treated with a single 100 μg/kg subcutaneous injection of PEG-rhG-CSF; P + R, treated with a single 50 μg/kg subcutaneous injection of PEG-rhG-CSF and 5 μg/kg/day rhG-CSF; RC, treated with 5 μg/kg/day rhG-CSF. PEG, pegylated; rhG-CSF, recombinant human granulocyte colony-stimulating factor. There were pairwise significant differences after 36 h among the three groups of patients treated with PEG-rhG-CSF (P<0.05); the P-50 and P + R groups exhibited no significant differences (P>0.05); the P-50 and P+R groups exhibited statistically significant difference from the P-100 group (P<0.05). The values show the mean ± standard deviation.

**Table III:** Remission time comparison of neutropenia-induced symptoms after therapy in the patients with grade IV neutropenia in the experimental and control groups.

Symptoms	Group	n	Remission time (h)	F-statistic	P-value
Fever	Experimental	63	30.00±7.48	85.79	<0.01
	Control	12	72.00±17.89		
Weakness	Experimental	64	66.00±11.14	2.12	0.10
	Control	17	78.00±11.56		
Skeletal muscle pain	Experimental	51	30.00±5.10	81.11	<0.01
	Control	10	59.00±11.46		
Digestive tract symptoms	Experimental	33	66.00±11.14	3.11	0.10
	Control	8	78.00±11.56		

The values for the remission time show the mean ± standard deviation.
